# Highly Differentiated ZW Sex Microchromosomes in the Australian *Varanus* Species Evolved through Rapid Amplification of Repetitive Sequences

**DOI:** 10.1371/journal.pone.0095226

**Published:** 2014-04-17

**Authors:** Kazumi Matsubara, Stephen D. Sarre, Arthur Georges, Yoichi Matsuda, Jennifer A. Marshall Graves, Tariq Ezaz

**Affiliations:** 1 Institute for Applied Ecology, University of Canberra, Canberra, ACT, Australia; 2 Department of Applied Molecular Biosciences, Graduate School of Bioagricultural Sciences, Nagoya University, Nagoya, Aichi, Japan; University of Florence, Italy

## Abstract

Transitions between sex determination systems have occurred in many lineages of squamates and it follows that novel sex chromosomes will also have arisen multiple times. The formation of sex chromosomes may be reinforced by inhibition of recombination and the accumulation of repetitive DNA sequences. The karyotypes of monitor lizards are known to be highly conserved yet the sex chromosomes in this family have not been fully investigated. Here, we compare male and female karyotypes of three Australian monitor lizards, *Varanus acanthurus*, *V. gouldii* and *V. rosenbergi*, from two different clades. *V. acanthurus* belongs to the *acanthurus* clade and the other two belong to the *gouldii* clade. We applied C-banding and comparative genomic hybridization to reveal that these species have ZZ/ZW sex micro-chromosomes in which the W chromosome is highly differentiated from the Z chromosome. In combination with previous reports, all six *Varanus* species in which sex chromosomes have been identified have ZZ/ZW sex chromosomes, spanning several clades on the varanid phylogeny, making it likely that the ZZ/ZW sex chromosome is ancestral for this family. However, repetitive sequences of these ZW chromosome pairs differed among species. In particular, an (AAT)n microsatellite repeat motif mapped by fluorescence *in situ* hybridization on part of W chromosome in *V. acanthurus* only, whereas a (CGG)n motif mapped onto the W chromosomes of *V. gouldii* and *V. rosenbergi*. Furthermore, the W chromosome probe for *V. acanthurus* produced hybridization signals only on the centromeric regions of W chromosomes of the other two species. These results suggest that the W chromosome sequences were not conserved between *gouldii* and *acanthurus* clades and that these repetitive sequences have been amplified rapidly and independently on the W chromosome of the two clades after their divergence.

## Introduction

Sex-determining systems are highly divergent between different lineages of reptiles and birds [1]–[4]. In particular, three modes of sex determination, genotypic sex determination (GSD), male heterogamety (XX/XY) and female heterogamety (ZZ/ZW), including systems with multiple sex chromosomes, and temperature-dependent sex determination (TSD), have a haphazard distribution across the phylogeny in squamate reptiles [3], [5], [6]. This suggests that transitions between sex determination systems have occurred in many lineages of squamates [4], [7] and it follows that novel sex chromosomes will have arisen also multiple times with those transition. The degrees of heteromorphism between sex chromosomes differ among species [6] but little is known about how sex chromosomes form and differentiate in reptiles. In particular, the morphology and degree of Y or W degeneration show major differences even within a single taxonomic group. For example, Z and W chromosomes of *Gehyra purpurascens* (Gekkonidae) have different morphologies between different races [8], the Y chromosomes of three legless lizards (Pygopodidae) have distinctively different morphologies [9] and the W chromosomes of snakes show various levels of degeneration compared with a conserved Z chromosome [10], [11]. Y and W chromosomes are predisposed to accumulate repetitive DNA sequences by suppression of recombination with their counterparts, X and Z chromosomes, and such accumulation of repetitive DNA sequences promotes further differentiation between sex chromosomes [12]. Thus the accumulation of repetitive DNA sequences is thought to be an important step in sex chromosome differentiation.

Lizards from the family Varanidae are one group of squamates for which the karyotypes appear highly conserved. This family, which comprises a single extant genus *Varanus*, incorporating seventy-three species is widely distributed through Africa, western, central, southern and southeastern mainland Asia, Sri Lanka, Malaysian and Indonesian islands, islands of the Indian Ocean and the South China Sea, Philippines, Papua New Guinea and Australia [13]. Molecular phylogenetic studies have showed that the genus *Varanus* is divided into three major clades, African species, Asian species and Australian (Oceania) species [14]–[18]. In the latest study, the phylogeny of 39 *Varanus* species was analyzed using 1914-bp nuclear and 1995-bp mitochondrial gene sequences [18]. The result showed that the genus *Varanus* was divided into seven smaller clades, *niloticus* (African), *salvator* (Asian), *indicus* (Asian), *varius* (Australian) *gouldii* (Australian), *tristis* (Australian), and *acanthurus* clades (Australian).

Karyotypes have been reported for 22 *Varanus* species, four from the *niloticus* clade, five from the *salvator* clade, one from the *indicus* clade, one from the *varius* clade, five from the *gouldii* clade, three from the *tristis* clade and three from the *acanthurus* clade [19]–[21]. All species examined so far have an identical chromosome number, 2n = 40 consisting of 16 macrochromosomes and 24 microchromosomes, indicating that the karyotypes at this level are highly conserved in this family. This high conservation of karyotypes makes varanid lizards an excellent model to examine the fine scale molecular evolution of DNA sequences on some local chromosomal regions. Sex chromosomes are identified in four species only – two species from *niloticus* clade (*V. albigularis* and *V. niloticus*), one species from *varius* clade (*V. varius*) and one species from *acanthurus* clade (*V. acanthurus*). All four species have ZZ/ZW micro sex chromosomes in which the W is distinctively larger than other microchromosomes [19], [20]. The sex chromosomes of the many remaining species are unknown, and the molecular composition and constitution of the W chromosomes are yet to be investigated in any species.

In this study, we extend the comparison of sex chromosomes at the molecular level to additional varanid species. We examined the karyotypes of three *Varanus* species, the ridge-tailed monitor (*V. acanthurus*), the sand goanna (*V. gouldii*) and the heath monitor (*V. rosenbergi*). The first species belongs to the *acanthurus* clade and the remaining two belong to the *gouldii* clade [18]. Thus these three species gave us opportunity to examine molecular evolution on sex chromosomes between closely related species. We identified sex microchromosomes of these species using C-banding and comparative genomic hybridization. In addition, we conducted chromosome mapping of eighteen microsatellite motifs to chromosomes of these species. Finally, we prepared chromosome probes from *V. acanthurus* microchromosomes including W chromosome and carried out cross-species chromosome painting to the other two species. We use those results to infer the evolution of sex chromosomes in these species.

## Materials and Methods

### Animals

A male and a female of two monitor lizards, *Varanus acanthurus* and *V. gouldii*, were purchased from commercial suppliers. A male and a female *V. rosenbergi* were hatched in captivity from eggs collected for other studies from Kangaroo Island, South Australia. We identified the species with the aid of keys [22].

### Ethics Statement

Animal care and experimental procedures were performed following the guidelines of the Australian Capital Territory Animal Welfare Act 1992 (Section 40) and conducted under approval of the Committee for Ethics in Animal Experimentation at the University of Canberra (Permit Number: CEAE 11/07).

### Cell Culture and Chromosome Preparation

The tail tips were cut from each animal and used for cell culture. Metaphase chromosome spreads were prepared from fibroblasts of tail tissue following the protocol described in Ezaz et al. [23]. Briefly, minced tail tissues were implanted in a T25 culture flask containing AmnioMax medium (Life Technologies, Carlsbad, California, USA) and were allowed to propagate under the condition of at 28°C and in an atmosphere of 5% CO_2_. Once the fibroblasts had grown to about 80% confluency, cultures were split intoT75 flasks and subsequently split up to four passages before the chromosomes were harvested. Colcemid (Roche, Basel, Switzerland) was added to the culture flask to a final concentration of 75 ng/ml prior to harvesting. Cultured cells were harvested by trypsin treatment, suspended in 0.075 M KCl, then fixed in 3∶1 methanol:acetic acid and the cell suspension dropped onto glass slides and air-dried.

### C-banding

The C-banded chromosomes were obtained by the CBG method (C-bands by Barium hydroxide using Giemsa) [24], [25]. Slides were soaked in 0.2 N HCl for 40 min and rinsed with distilled water. Chromosomes were denatured in 5% Ba(OH)_2_ for 5 min at 50°C. Denaturation was stopped by rinsing the slides in 0.2 N HCl and distilled water, then chromosomes were renatured by incubation in 2×SSC (Saline Sodium Citrate) for 60 min at 60°C. Then the slides were rinsed by distilled water and stained with 4% Giemsa for 30 min.

### DNA Extraction and Synthesis of Microsatellite DNA Probe

Total genomic DNA was extracted from cultured fibroblasts using the DNeasy kit (Qiagen, Netherlands) and following the manufacturer protocols. Cy3-labeled oligonucleotides of 18 microsatellite motifs – (AC)_15_, (AG)_15_, (AT)_15_, (AAC)_10_, (AAT)_10_, (AGC)_10_, (CGG)_10_, (GAG)_10_, (AAAC)_8_, (AAAT)_8_, (AAGG)_8_, (AATC)_8_, (AATG)_8_, (ACGC)_8_, (AGAT)_8_, (ATCC)_8_, (AAAAT)_6_ and (AAATC)_6_ – were purchased from GeneWorks (Hindmarsh, South Australia, Australia).

### Microdissection and Preparation of Chromosome Probes

We performed microdissection using an inverted phase contrast microscope Zeiss Axio vert.A1 (Zeiss, Oberkochen, Germany) equipped with Eppendorf TransferMan NK 2 micromanipulator (Eppendorf, Hamburg, Germany). Glass needles were made from 1.0 mm diameter capillary glass using a glass capillary puller, Sutter P-30 Micropipette Puller (Sutter Instrument, Novato, California, USA) and sterilized by irradiation of ultra violet. Microchromosomes including W chromosome were scratched from freshly prepared chromosome slides of female *V. acanthurus* with a glass needle using the micromanipulation system and transferred into 0.2 ml PCR tubes. Chromosome DNAs were amplified using GenomePlex Single Cell Whole Genome Amplification Kit (Sigma-Aldrich, St. Louis, Missouri, USA) according to the manufacture’s protocol with slight modification. The volume for all reaction steps was scaled down to half. PCR cycle for amplification of DNAs was increased to 30.

### Comparative Genomic Hybridization (CGH) and Fluorescence In**Situ Hybridization (FISH)

CGH and FISH with microsatellite motifs probes were conducted using methods described in our previous study [9], [25]. For chromosome probes, we conducted FISH with slight modification. Chromosome probes were labelled by nick translation incorporating SpectrumGreen-dUTP (Abbott, North Chicago, Illinois, USA) or SpectrumOrange-dUTP (Abbott). Each labelled probe was precipitated with 20 µg glycogen as carrier, and dissolved in 15 µl hybridization buffer. The hybridization mixture was placed on a chromosome slide and sealed with a coverslip and rubber cement. Probe DNA and chromosome DNA were denatured by heating the slide on a heat plate at 68.5°C for 5 min. The slides were hybridized overnight in a humid chamber at 37°C. Hybridization was carried out for 2 days in cross-species chromosome painting. The slides were then washed by the following series: 0.4×SSC, 0.3% IGEPAL (Sigma-Aldrich) at 55°C for 2 min followed by 2×SSC, 0.1% IGEPAL at room temperature for 1 min. The slides were dehydrated by ethanol series and air-dried and then counterstained using 20 µg/ml DAPI (4′,6-diamidino-2-phenylindole), 2×SSC and mounted with anti-fade medium, Vectashield (Vector Laboratories, Burlingame, California, USA).

## Results

### Karyotyping

**Figure 1:**
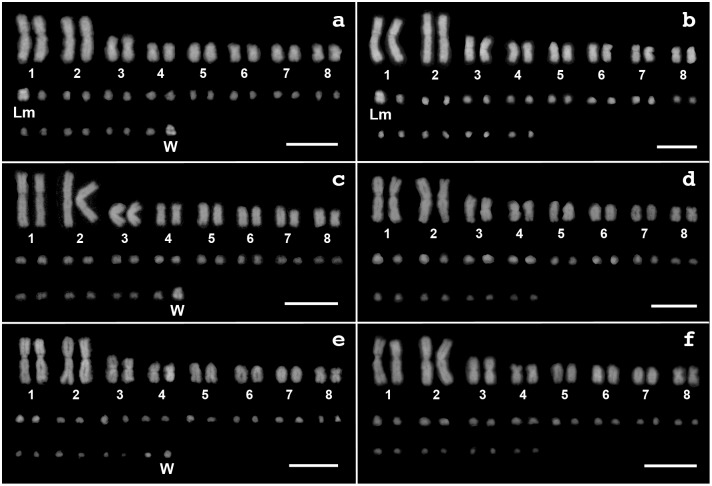
DAPI-stained karyotypes of three *Varanus* species. Female (a) and male (b) of *V. acanthurus*, female (c) and male (d) of *V. rosenbergi*, and female (e) and male (f) of *V. gouldii*. Macrochromosomes are numbered according to King and King [19] and King et al. [20]. ‘W’ and ‘Lm’ indicate W chromosomes in the three species (a, c, e) and large microchromosomes in male and female *V. acanthurus* (a, b), respectively. Scale bars indicate 10 µm. W chromosome in *V. gouldii* was identified by C-banding and CGH (Figure 2, 3).

DAPI-staining of the karyotypes identified the diploid number of chromosomes for all three *Varanus* species to be 2n = 40 (Figure 1). These karyotypes all consist of 16 macrochromosomes and 24 microchromosomes. The macrochromosomes are all bi-armed except for the acrocentric chromosome 5 in *V. acanthurus* (Figure 1a, b) and acrocentric chromosomes 5–7 in the remaining two species (Figure 1c–f).

Sex chromosomes were identified by their heteromorphism in females. The female karyotype of *V. acanthurus* has two large sized microchromosomes compared with the male karyotype which has only one (Figure 1a, b). The two large microchromosomes in the female karyotype have a different morphology to each other, one (indicated by ‘W’ in Figure 1a) is acrocentric and the other one (indicated by ‘Lm’ in Figure 1a) is metacentric, but one of the two (indicated by ‘Lm’ in Figure 1a) is morphologically similar to the large microchromosome in the male karyotype (indicated by ‘Lm’ in Figure 1b). It was previously reported that *V. acanthurus* has a ZZ/ZW sex chromosome system in which the acrocentric W chromosome is larger than other microchromosomes [20]. However, the presence of another large size microchromosome than W chromosome has not previously been reported. Thus, we conclude that the large metacentric microchromosome observed in both female and male used in this study is a polymorphism of an autosome or Z chromosome. In *V. rosenbergi*, comparison of the karyotypes between male and female showed that a large microchromosome is present in female but not in male, so is presumed to be a W chromosome (Figure 1c, d). No large size microchromosomes were observed in the karyotypes of *V. gouldii* (Figure 1e, f) although W chromosome was identified by C-banding and CGH as we describe later.

### C-banding

**Figure 2:**
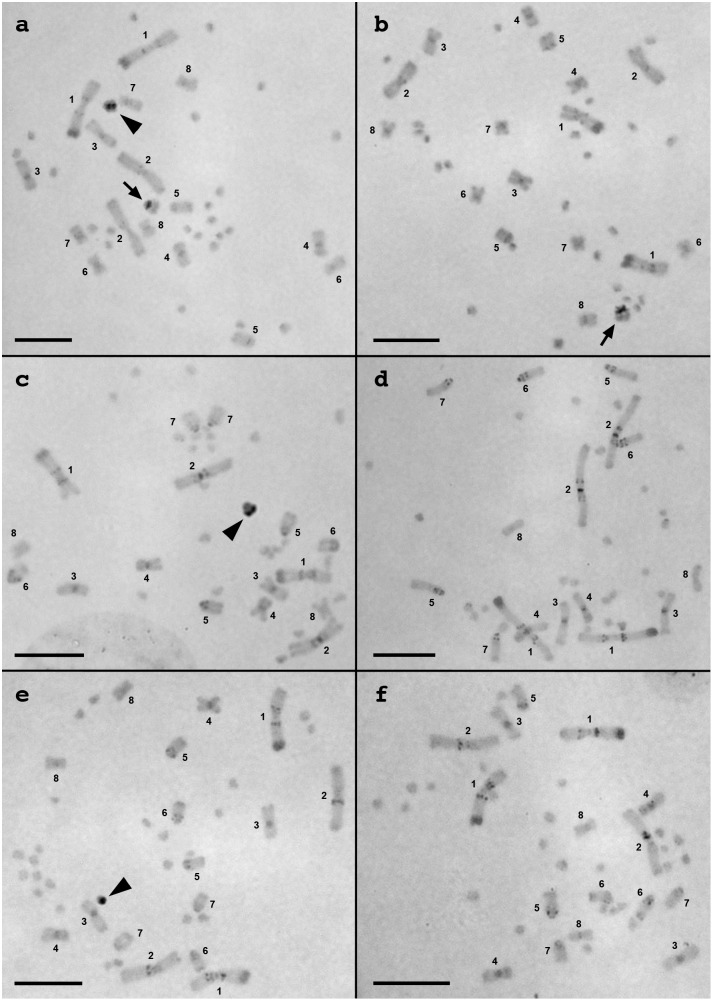
C-banded metaphase spreads of three *Varanus* species. Female (a) and male (b) of *V. acanthurus*, female (c) and male (d) of *V. rosenbergi*, and female (e) and male (f) of *V. gouldii*. Macrochromosomes are numbered. Arrowheads and arrows indicate W chromosomes in the three species (a, c, e) and large microchromosome in male and female *V. acanthurus* (a, b), respectively. Scale bars indicate 10 µm.

C-bands were detected at the centromeric regions of almost all chromosomes, on interstitial regions of chromosome pairs 1, 2 and 5, and on the telomeric regions of chromosome 1q in the three species (Figure 2). Small C-bands were also detected on proximal regions of chromosome pairs 6 and 7 in *V. rosenbergi* and *V. gouldii* (Figure 2c–f).

In addition to these autosomal C-bands, an intense C-band was detected on a single microchromosome in females, but not in males, of the three species (Figure 2). This implies that the three *Varanus* species have ZZ/ZW sex chromosomes and their W chromosomes are highly heterochromatic. The W chromosomes correspond to a large microchromosome in DAPI-stained karyotypes of *V. acanthurus* and *V. rosenbergi* (Figure 1a, c). A smaller but intense C-band was also detected on the short arm of large metacentric microchromosome in both female and male of *V. acanthurus* (Figure 2a, b).

### CGH

**Figure 3:**
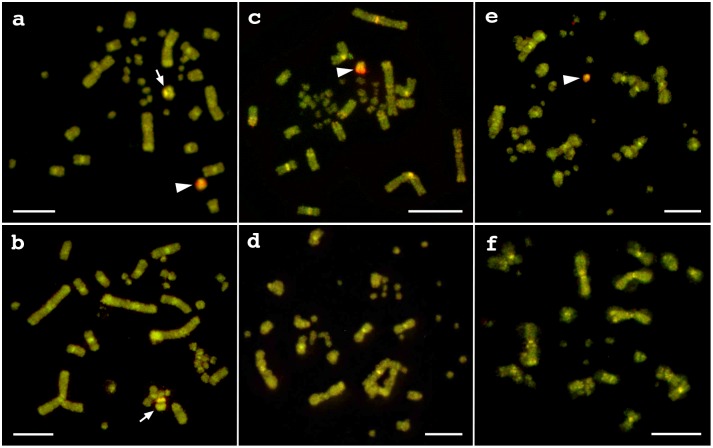
Comparative genomic hybridization (CGH) images of three *Varanus* species. Female (a) and male (b) of *V. acanthurus*, female (c) and male (d) of *V. rosenbergi*, and female (e) and male (f) of *V. gouldii*. Arrowheads and arrows indicate W chromosomes (a, c, e) and large size microchromosome in male and female *V. acanthurus* (a, b), respectively. Scale bars indicate 10 µm.

CGH images showed a bright hybridization signal produced by female genomic DNA in metaphase spreads in females, but not in males, of the three species (Figure 3), implicating a female-specific W chromosomes. These results confirm that the three *Varanus* species all have ZZ/ZW sex chromosomes in which W chromosome is easily identified but that the Z chromosomes are not distinguishable from the autosomes by CGH. In *V. acanthurus*, there was hybridization signal on large metacentric microchromosome that did not show female- or male-bias.

### FISH Mapping of Microsatellite Motifs

**Figure 4:**
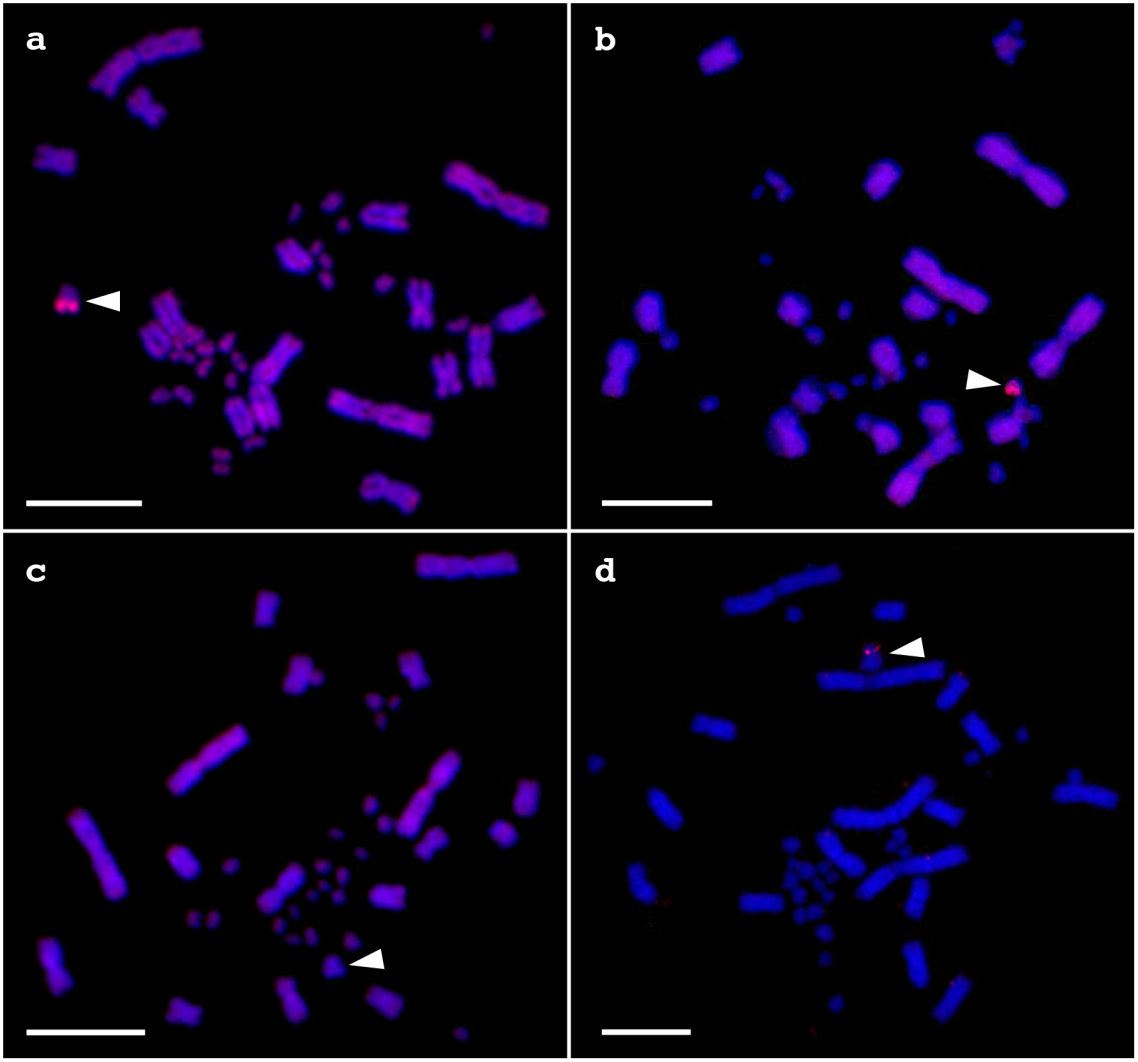
FISH mapping of microsatellite motifs in three *Varanus* species. FISH mapping of (CGG)_10_ microsatellite motif in female *V. rosenbergi* (a), female *V. gouldii* (b), female *V. acanthurus* (c), and of (ATT)_10_ microsatellite motif in female *V. acanthurus* (d). Arrowheads indicate W chromosomes (a–d). Fluorescent signal of (CGG)_10_ microsatellite motif was not observed on W chromosome of *V. acanthurus* (c). Scale bars indicate 10 µm.

We examined the chromosome distribution of 18 microsatellite motifs by FISH in females of the three *Varanus* species. Two of the 18 were mapped onto the sex chromosomes. Results were inconsistent between species. The (CGG)_10_ motif showed hybridization signals on all chromosomes in all the three species and also bright hybridization signals on the W chromosomes of *V. rosenbergi* and *V. gouldii* (Figure 4a, b) but not on the W chromosome of *V. acanthurus* (Figure 4c). This suggests that W chromosomes of *V. rosenbergi* and *V. gouldii* contain an extensive amplification of the CGG microsatellite repeat. In contrast, the repeat motif (AAT)_10_ showed intense hybridization signal on the proximal region of the W chromosome in the female metaphase of *V. acanthurus* but not on other regions (Figure 4d). Specific hybridization signal by this probe was not observed on metaphases in *V. rosenbergi* and *V. gouldii* (Data not shown).

### Chromosome Painting with V. Acanthurus Microchromosome Probes

We prepared 32 chromosome probes from *V. acanthurus* microchromosomes. The first and second ones were amplified from the W chromosome and the large microchromosome, respectively. These two chromosomes were easily distinguishable from each other and other microchromosomes so that we collected six W chromosomes and five the large microchromosomes from multiple metaphases into each one PCR tube and amplified the chromosome DNAs (data not shown). The other microchromosomes could not be distinguished from each other. For this reason, the other 30 probes were amplified from single microchromosomes randomly collected from eight metaphases.

**Figure 5:**
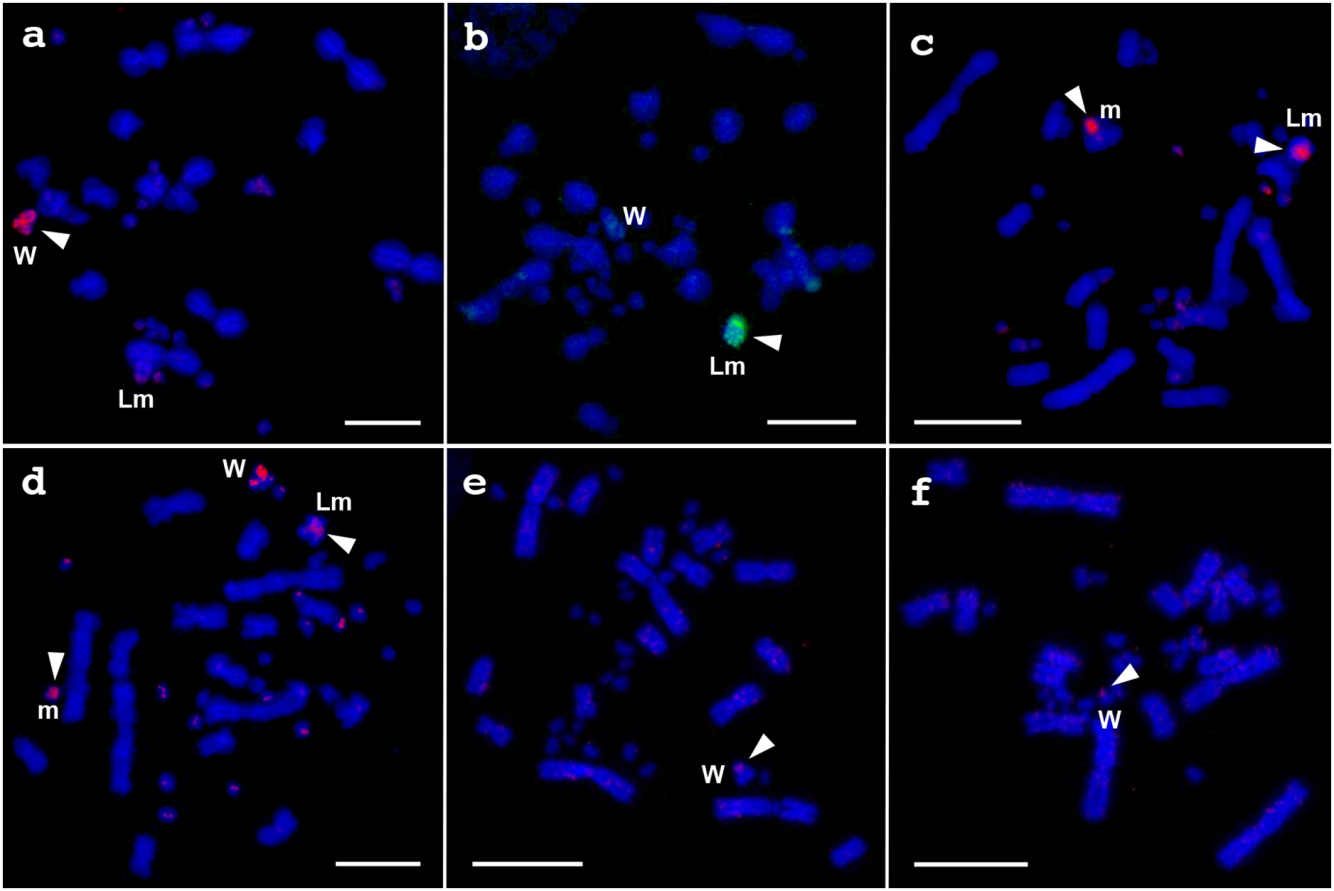
Chromosome painting with *V. acanthurus* microchromosome probes. Painting with the W chromosome probe in female *V. acanthurus* (a), the large microchromosome probe in female *V. acanthurus* (b), an autosomal microchromosome probe in male (c) and female *V. acanthurus* (d), and the W chromosome probe in female *V. rosenbergi* (e) and *V. gouldii* (f). Arrowheads indicate hybridization signals. ‘W’, ‘Lm’ and ‘m’ indicate W chromosomes in the three species (a, b, d–f), large microchromosomes in male and female *V. acanthurus* (a–d), and microchromosome to which the probe has been hybridized in male and female *V. acanthurus* (c, d), respectively. Scale bars indicate 10 µm.

We carried out chromosome painting with the 32 probes to metaphase spreads of *V. acanthurus* to test their painting patterns. The W chromosome probe produced bright hybridization signals on the whole region of W chromosome and weak signals on some microchromosomes (Figure 5a). The large microchromosome probe produced bright hybridization signals on the whole region of large microchromosome and weak signals on the centromeric region of the short arm and on the telomeric region of the long arm of chromosome 2 (Figure 5b). Four of the other 30 probes did not show specific painting signals on any microchromosomes (data not shown). One of the remaining 26 probes showed intense hybridization signals on four pairs of microchromosomes and weak hybridization signals on the W chromosome (data not shown). All of the remaining 25 probes produced painting signals on a single pair of microchromosomes. All of the 25 probes were hybridized not only on themselves but also on W chromosome and many of other microchromosomes (e.g. Figure 5c, d), indicating that W chromosome shared repetitive sequences with other microchromosomes. One of the 25 probes was hybridized on a normal size microchromosome and the large microchromosome in both male and female metaphase spreads (Figure 5c, d). This result indicates that the large microchromosome is a polymorphism of an autosomal microchromosome.

Cross-species chromosome painting with the *V. acanthurus* W chromosome probe showed weak hybridization signals on the centromeric region of W chromosomes in the female *V. rosenbergi* and *V. gouldii* (Figure 5e, f).

## Discussion

We found the total chromosome number, number of macro and micro-chromosomes and morphologies of macrochromosomes of the six individuals from three species examined in this study were identical to those reported previously [19], [20] confirming that at a gross morphological level, the karyotypes of *Varanus* species are conservative. However, we also identified a large polymorphic microchromosome in *V. acanthurus* that had not previously been reported. This large metacentric microchromosome is a polymorphism of an autosomal microchromosome and may have been enlarged by the accumulation of repetitive sequences on the short arm.

**Figure 6:**
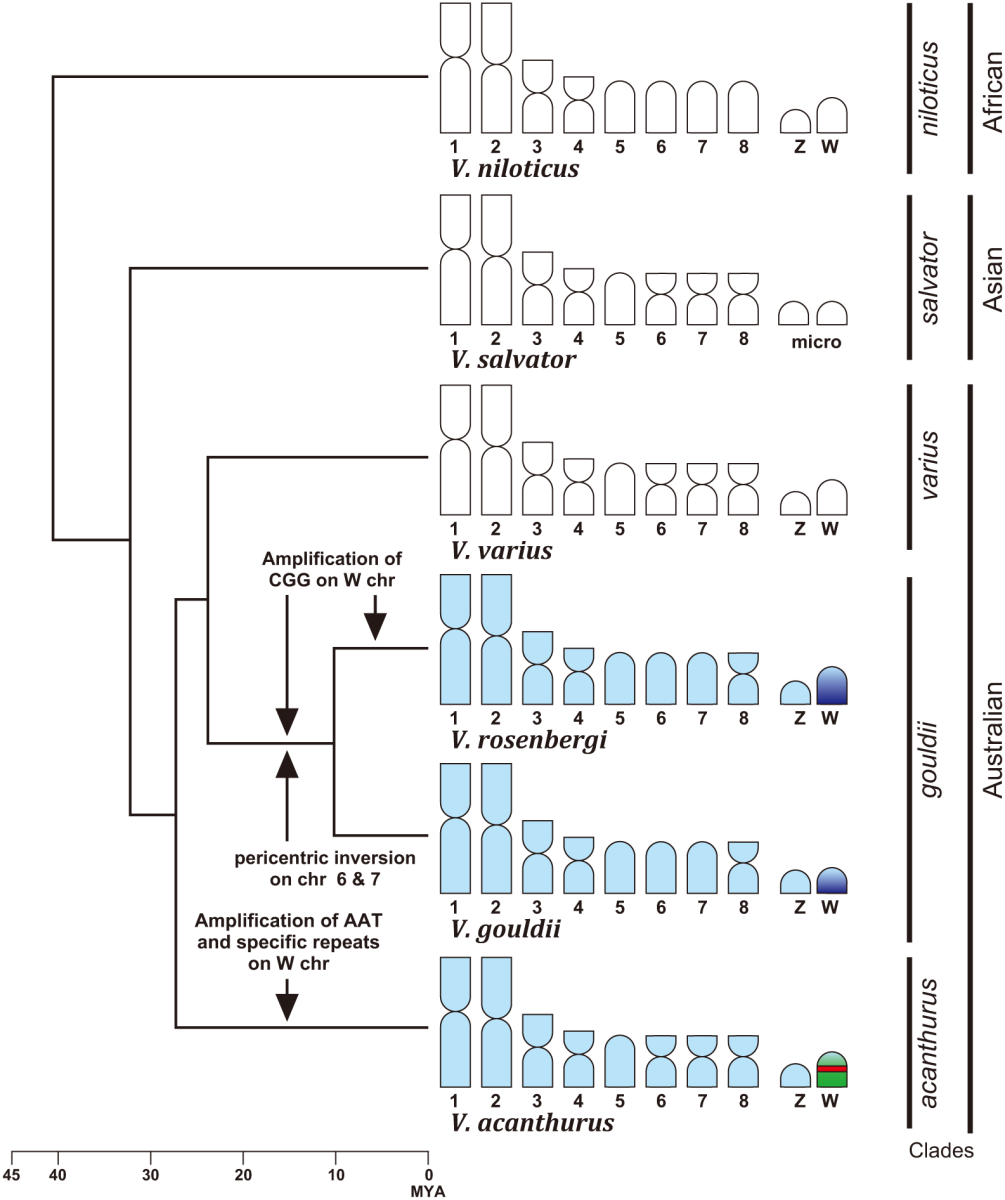
Schematic model for karyotype and sex chromosome evolution in varanid species. Phylogeny and clade names are referred to Vidal et al. [18]. Divergence times were estimated to be ca. 41, 32, and 27 million years ago (MYA) for nodes between African species and rests, between Asian and Australian species, and among the three clades in Australian species, respectively [18]. Karyotypes of *V. niloticus* (*niloticus* clade), *V. salvator* (*salvator* clade) and *V. varius* (*varius* clade) are referred to King and King [19]. The CGG repeat motif (light and dark blue) was widely distributed over the genome of the common ancestor of *acanthurus* and *gouldii* clades, and, then, was rapidly amplified on the W chromosome in the common ancestor of *V. gouldii* and *V. rosenbergi*. Further amplification of the CGG repeat motif occurred on the W chromosome in *V. rosenbergi*. The AAT repeat motif (red) and other specific repetitive sequences (green) were independently accumulated and amplified on the W chromosome in *V. acanthurus*. Pericentromeric inversions had occurred on chromosomes 6 and 7 in the common ancestor of *V. gouldii* and *V. rosenbergi*.

A comparison among karyotypes of the three species reveals other distinct morphological differences between the *acanthurus* clade (represented by *V. acanthurus*) and the *gouldii* clade (represented by *V. gouldii* and *V. rosenbergi*), where chromosomes 6 and 7 are bi-armed in the former and acrocentric in the latter clade (Figure 1). *V. varius* (the *varius* clade) and two species from the *salvator* clade, *V. bengalensis* and *V. salvator*, have similar karyotypes with *V. acanthurus*; chromosomes 6 and 7 are bi-armed in the three species (Figure 6) [19]. This suggests that pericentromeric inversions had occurred on chromosomes 6 and 7 in the *gouldii* clade (Figure 6). However, chromosomes 6 and 7 are acrocentric in *V. exanthematicus* and *V. niloticus* (Figure 6) [19], both from the *niloticus* clade which was diverged first from the common ancestor of extant varanids [14]–[18]. Recently, comparisons of gene locations among *V. salvator* (*salvator* clade), *V. exanthematicus* (*niloticus* clade), an agamid (*Leiolepis reevesii rubritaeniata*) and a snake (*Elaphe quadrivirgata*) revealed that chromosome 6–8 of *V. exanthematicus* retained the gene orders inherited from their common ancestor whereas some intrachromosomal rearrangements probably occurred on chromosome 6–8 of *V. salvator* [26]. Thus an alternative scenario in which the inversions have occurred on the ancestral acrocentric chromosomes 6 and 7 in the *varius* and the *acanthurus* clades might still be possible. Molecular cytogenetic studies involving representatives of each clade and including appropriate outgroup taxa will be necessary to infer the chromosome rearrangements in varanid lizards.

Our C-banding and CGH data identified conclusively that all three species have ZZ/ZW sex microchromosomes and the W chromosomes are highly heterochromatic with the accumulation of large amount of female specific DNAs during their differentiation from Z chromosomes. Although we could not obtain Z chromosome probes and therefore could not investigate homologies of Z chromosomes among species, we did show that the *V. acanthurus* W chromosome probe hybridized to the centromeric regions of W chromosomes in the other two species. This suggests that the sex chromosomes of the three species were probably derived from the one ancestral pair of chromosomes. An absence of hybridization signals on the Z chromosomes, suggest that the hybridization signals we did see on the W were probably from some repetitive sequences common to the W chromosomes of the two clades. The inclusion of the data reported here brings to six out of the six *Varanus* species that have been examined (from the *niloticus*, *acanthurus*, *varius* and *gouldii* clades) which exhibit ZZ/ZW sex chromosomes as a pair of microchromosome (Figure 6) [19]. This suggests that the common ancestor of the extant *Varanus* species also had a ZZ/ZW sex chromosome. However, the sex determination systems of most Asian species of *Varanus* have not yet been sufficiently investigated [27] so further study of homologies among the sex chromosomes of each clade will be required to confirm or refute this proposition.

Our FISH analyses with microsatellite motifs indicate that the (CGG)n repeat motif has been highly amplified on the W chromosome and has become the main component of the long arm of the W chromosome in *V. gouldii* and *V. rosenbergi*. In contrast, fluorescent signal from the repeat motif (CGG)n was not detectable on the W chromosome of *V. acanthurus*. Instead, a different microsatellite repeat motif, (AAT)n, was mapped with intense signals on the proximal region of the W chromosome in *V. acanthurus*. Furthermore, *V. acanthurus* W chromosome probe produced hybridization signals only on the centromeric regions of W chromosomes of the other two species. These results suggest that most sequences are not conserved between the W chromosomes of the two clades, *gouldii* and *acanthurus* clades, that diverged around 27 million years ago [18]. The repeat motif CGG showed hybridization signal across the entire karyotype in all three species, suggesting that this motif was widely distributed across the genome of the common ancestor of the two clades, but subsequently was rapidly amplified on the W chromosome in the *gouldii* clade after it diverged from other clades (Figure 6). This process of differential amplification of the (CGG)n repeat motif has also continued within the *gouldii* clade with the W chromosome of *V. rosenbergi* being comparatively larger than that of *V. gouldii*. On the other hand, the (AAT)n repeat motif and other specific repetitive sequences were probably amplified on the W chromosomes in *V. acanthurus* independent from the *gouldii* clade (Figure 6). The W chromosomes are distinctively larger than the Z chromosomes in *V. albigularis*, *V. niloticus* and *V. varius* [19] as well as those of *V. acanthurus* and *V. rosenbergi* studied here, so it is likely that the W chromosomes are highly differentiated from their Z partners in all varanid lizards. It will be interesting to widen this study to molecular cytogenetic studies of African and Asian species to infer the evolution of the sex chromosomes in varanid lizards.

Our comparative study of sex chromosomes in the monitor lizards presents direct evidence that rapid evolution of repeat sequences is associated with the differentiation of sex chromosomes. The accumulation of repetitive sequences has frequently occurred on the sex chromosomes in various animals and plants [28]–[35]. Whether this accumulation of repetitive sequences has initiated other associated mechanisms, such as suppression of recombination, is yet to reveal through further studies including linkage mapping and high resolution comparative analysis of sex chromosomes at sequence level. In addition, comparative studies of repetitive sequences on sex chromosomes in diverse taxa will provide further molecular evidence about the mechanism behind evolution and degeneration of sex chromosomes.
